# A hidden pattern? Common polyextremophilic microbial adaptations in Icy Moon analog environments

**DOI:** 10.3389/fspas.2026.1786844

**Published:** 2026-04-20

**Authors:** Sandra Gusi-Martínez, Alberto G. Fairén, Miguel Ángel Fernández-Martínez

**Affiliations:** 1 Molecular Biology Laboratory, CBRN Defence Systems Department, Campus La Marañosa, Instituto Nacional de Técnica Aeroespacial “Esteban Terradas” (INTA), Madrid, Spain; 2 Centro de Astrobiología (CAB), CSIC- INTA, Madrid, Spain; 3 Department of Astronomy, Cornell University, New York, NY, United States; 4 Department of Ecology, Universidad Autónoma de Madrid, Madrid, Spain; 5 Centro de Investigación en Biodiversidad y Cambio Global (CIBC-UAM), Universidad Autónoma de Madrid, Madrid, Spain

**Keywords:** cryoenvironments, cryophiles, extraterrestrial analogs, halophiles, meta-analysis, subsurface environments

## Abstract

The search for life signatures beyond Earth is one of the main objectives of space exploration. Studies of analogous terrestrial ecosystems have shed light on the limits of life and on the adaptations of microbial communities to thrive in these extreme environments resembling Icy Moons. However, their findings tend to be compartmentalized, which hinders the drawing of broad conclusions about the drivers and challenges for life. This study aims to identify general characteristics of microbial communities inhabiting terrestrial analogs of Icy Moons, applying a novel meta-analysis on publicly available *16S rRNA* amplicon sequencing data. We also seek to apply our findings to a new fundamental approach in the search for life in Europa and Enceladus, locations where life may exist. Our results suggest that depth, pH and hypersalinity are the key environmental drivers for microbial taxa distribution and molecular adaptations, with halophilic archaea showing ubiquitous presence. Integrating diverse datasets into a single meta-analysis allowed us to infer statistically significant microbial patterns related to adaptation to the Icy Moons’ analog conditions, notably that osmolytes and modified lipids emerged as a shared adaptive strategy, regardless of depth. Our findings are aimed to helping guide future life detection efforts in these extraterrestrial environments.

## 1 Introduction

**Figure 1:**
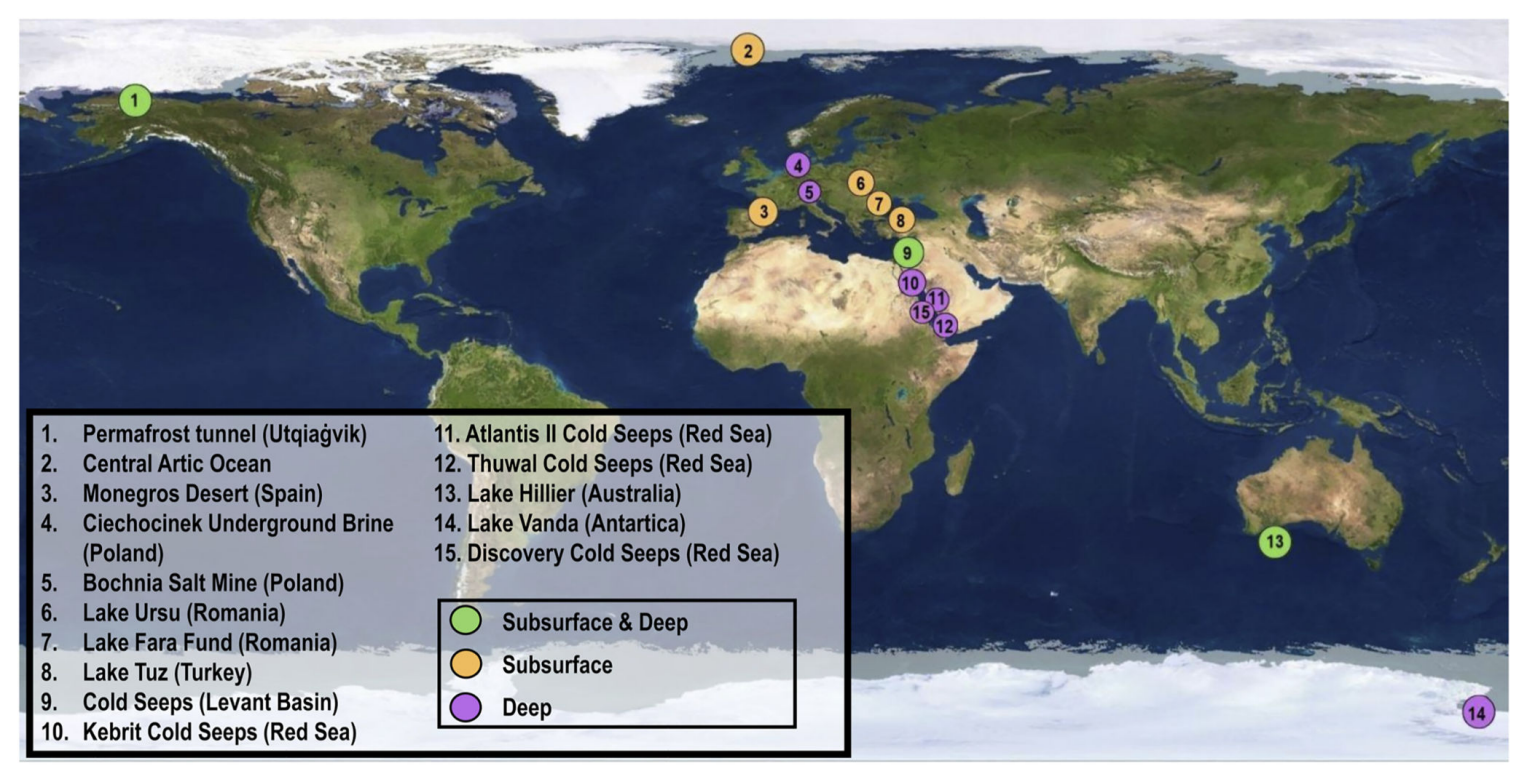
Geographic map showing the sampling locations and corresponding classification of the analyzed samples.

Since late 1990s, the possibility of conditions compatible with life occurring on extraterrestrial Solar System planetary bodies, such as the Icy Moons of Jupiter (Europa, Ganymede and Callisto) and Saturn (Tetis, Dione, Rhea, Mimas, Enceladus and Iapetus) has been seriously considered (Nimmo and Pappalardo, 2016; Grasset et al., 2012). These theories arose after NASA’s and ESA’s orbital missions, such as Galileo (active from 1995 to 2003) or Cassini (1997-2017) sent back the first scientific data and images of these moons at the beginning of the twenty-first century. These space probes detected subsurface liquid water (oceans) in contact with a rocky bottom, compounds essential for life (the so-called CHNOPS), other organic compounds, and chemical electron donor-electron acceptor pairs in Europa and Enceladus (Kang et al., 2022; Coelho and Martins, 2021; Cockell et al., 2021; Cockell et al., 2024; Nimmo and Pappalardo, 2016; Postberg et al., 2018; Gaidos et al., 1999). However, there is still the need to confirm and broaden these findings by more extensive orbital and landing missions: to date, we only have an approximate description of surface and subsurface environments on these two satellites (Figure 1). Missions such as the JUICE probe, launched by the European Space Agency (ESA) in April 2023, or the “Ocean Worlds Exploration Program”, proposed by NASA for the decade 2033-2042 (Howell et al., 2021) are intended to fill those knowledge gaps.

In detail, data obtained so far from flyby missions show Europa, Jupiter’s smallest moon, has a thin atmosphere with a vertical oxygen density of approximately 2.4-1414 cm^-2^, CO_2_ and H_2_O that fluctuates depending on the face of the moon (De Kleer et al., 2023; Vance et al., 2018; Hall et al., 1995). This gaseous envelope surrounds a surface layer of salt-bearing ice between 5 and 30 km thick (Supplementary Figure S1). The fractures present in this outer ice cover revealed the presence of an underlying ocean with twice the mass of the terrestrial ocean and depths of over 100 km (Bagenal and Dols, 2020). Analysis of the water found in these areas has detected the presence of compounds (e.g., NaCl, MgSO_4_, other sulfur salts, etc.) probably originating from hydrogeochemical reactions occurring in hydrothermal vents present at the interface between the water and the seafloor (Coelho and Martins, 2021). pH could only be estimated within a range of acidic values (<7) for ocean water (Weber et al., 2023; Daswani et al., 2021; Tan et al., 2021).

On the other hand, Enceladus, the second smallest moon of Saturn, also presents a faint atmosphere, including water vapor, MgSO_4_, NH_3_, putative phosphate compounds, CO_2_ and other various organic molecules, such as monocyclic aromatic compounds and CH_4_ (Khawaja et al., 2025), which surrounds a surface layer of ice of approximately 40 km thick. Part of these atmospheric compounds are alleged to be related to plumes of water vapor, icy particles, and organic compounds that are ejected by tidal pumping from the subsurface ocean usually in the south polar region (Spencer et al., 2018). In the case of Enceladus, this ocean has been recently estimated to be 40 km deep (Ames et al., 2025). Based on the study of the abundance of CO_2_ present in the gas derived from the water columns, the pH of the underlying water has been estimated to be basic, with a range between 10.1-11.6 (Glein and Truong, 2025).

Although there is some environmental variation between these Icy Moons, the presence of hypersaline brines is a common hypothesized feature for both of them (Wolfenbarger et al., 2022; Castillo-Rogez et al., 2022; Fox-Powell and Cousins, 2020; Pappalardo et al., 2009). This idea is supported by Earth’s ocean ice dynamics knowledge, which has shown that a formation of brine channels occurs within the ocean-ice interface (Fox-Powell and Cousins, 2020), resulting in hypersaline environments within the ice and in the lower layer in contact with ocean water. For Europa, the ocean composition is estimated to be above 50 Practical Salinity Units (PSU) (Kang et al., 2022), ~1.5 times compared to the average salinity of the terrestrial open ocean, 35 PSU, (Suryanarayanan and Ravishankar, 2023; Millero et al., 2008), but similar to other terrestrial polar lakes or springs (Don Juan pond, Lake Vanda, Lake Untersee, etc., in Antarctica; Gypsum Hill, Lost Hammer springs in the High Arctic, Lay et al., 2012; Magnuson et al., 2022). Nonetheless, precise conditions for Enceladus’ ocean regarding its salinity are still being discussed.

Despite the knowledge about many environmental characteristics of Europa and Enceladus is still limited, both Icy Moons are assumed to harbor habitable environments, with possible counterparts in terrestrial analogs. Extreme terrestrial environments such as hypersaline lakes (e.g., lake Vanda in Antarctica or lake Hillier in Australia), deep marine hypersaline anoxic basins (e.g., Discovery or Kebrit in the Eastern Mediterranean Sea), and cold seeps (such as Haima in South China Sea or Amon mud volcano in the Nile deep-sea fan) are of particular interest in the search for life adapted to the physicochemical conditions likely present on the Icy Moons, as their salinity and underlying metallic or silicate cores are considered key features in defining Earth analogs for these extraterrestrial settings (Magnuson et al., 2022; Antunes et al., 2020; Jebbar et al., 2020; Pettinelli et al., 2015). However, terrestrial analogs to the Icy Moons explored so far are variable in many of their ecological parameters, thus making it difficult to state strong general conclusions. Consequently, this leads to hypothesize whether there will be recognizable, common, statistically significant compositional patterns for the microbial communities inhabiting them that could be used to postulate how Icy Moons’ putative organisms might look like.

The analysis of *16S rRNA* gene libraries is currently regarded as a comprehensive, high-resolution approach to characterizing microbial distribution patterns and their relationships with ecological environmental factors. This is because it overcomes the limitations of cultivation-based methods (Shuikan, et al., 2023; Shu and Huang, 2021). In the context of extreme environments, the use of sequencing techniques has enabled the identification of numerous lineages that were previously overlooked, thereby enhancing our understanding of the evolution of life and the ecological role of microorganisms (Shu and Huang, 2021). Hence, this study is devoted to identifying patterns in the microbial diversity through *16S rRNA* gene sequencing among several hypersaline terrestrial analogs of Europa and Enceladus that can be of interest for future life beyond Earth exploration missions, helping in a better focus for specific life signatures. To this end, information about possible biomarkers associated with metabolisms and adaptability to extreme environment factors that could be extrapolated from our results is also discussed.

## 2 Materials and methods

### 2.1 Data acquisition

We divided the terrestrial environments recognized as analogs of Icy Moons into two categories: The first group, “Subsurface”, was defined by those environments that could be influenced by atmospheric variables, such as radiation or deposition of organic matter, due to their proximity to the surface. We considered the “Subsurface” group as analogous to the frozen crust of the Icy Moons, where light penetration always exists even if it is very constrained, and pressure is not a limiting factor. The second group, “Depth”, was defined by environments isolated from atmospheric variables. We considered the “Depth” group as analogous to the liquid water subsurface oceans within the Icy Moons, since the frozen crust above isolates them from any external physical phenomenon. These characteristics create conditions of absence of light in the underlying waters, which are subjected to significantly greater pressure than that on the surface. For both categories, only environments with high salinity conditions (i.e., above 35 PSU were considered).

A thorough search of scientific articles focused on the description of the microbial communities in environments recognized as analogs of Icy Moons was performed using the Web of Science, Scopus and Google Scholar scientific databases. The keywords “hypersaline”, “microbiome” and “16s” were primarily used for the database search. This initial search returned 47 articles, which were classified based on the origin of the samples. Only those from non-surface environments were included in subsequent analyses, because the environmental conditions on Earth’s surface are completely different from those on the Icy Moons. A total of 42 studies met these requirements, of which 20 samples corresponded to “Subsurface” environments. This category included water samples from the euphotic zone, located up to 200 m below the surface of terrestrial water bodies (Jiao et al., 2014), as well as soil samples ranging between 0.10 and 0.25 m deep (Hao et al., 2021). Additionally, water samples from ponds within the ice layers of the Central Arctic Ocean were also included in this category, because light still passes through the ice and reaches the water below (Stroeve et al., 2021) what results in radiation-induced chemical changes that occur within the ice (Mifsud et al., 2022). On the other hand, the “Depth” group comprised 23 samples that were collected in aphotic zones either deeper than 200 m below the surface of water bodies (equivalent to a pressure of > 20 atm) or 0.25 m in terrestrial environments. As an exceptional case, samples from the bottom sediments of water bodies were included in the ‘Depth’ group, regardless of the distance to the surface as the overlying water column would stop and fragment charged particles present in space radiation (Kodaira et al., 2013).

After this initial classification, only publications that included *16S rRNA* gene amplicons sequenced with Roche 454 pyrosequencing, and Illumina Miseq or Hiseq platforms were selected, since these have a better performance and higher throughput in the sequencing of archaea and bacteria (D’Amore, et al., 2016). This filtering by sequencing method resulted in a total of 19 articles suiting these restricted parameters, of which 14 (32.56% of the initial number) were finally downloaded from the public database of the European Nucleotide Archive (ENA, https://www.ebi.ac.uk/ena/browser/), while the other 6 presented inconsistencies (Table 1; Figure 1; Supplementary Tables S1, S2). From these same studies, following what is already inferred for Europa and Enceladus and their terrestrial analogs, key environmental parameters (temperature, pH, depth) and salinity components (sulfate, calcium, phosphate, nitrate, nitrite, ammonia, magnesium, and sodium) were selected for statistical analyses (Supplementary Table S3). The substrate variable representing the physical matrix from which the samples were extracted (ice, sediment or water) was also included as a qualitative factor for ordination. All these environmental data were collected indistinctly from the metadata tables provided by the authors and deposited in the National Center for Biological Information, NCBI (https://www.ncbi.nlm.nih.gov/), or from data shown directly in the published articles. When lacking, this information was obtained from other research studies from the same sampling site.

### 2.2 Processing of *16S rRNA* gene reads

Each dataset was then processed independently by means of the DADA2 analysis package (Callahan et al., 2016; v. 3.17) in R (v. 4.3), in order to generate different amplicon sequence variants (ASVs) from the *16S rRNA* hypervariable regions (V3, V4, V6, V8 and V5) amplified in the selected studies. DADA2 package was chosen to perform the processing of reads and generation of ASVs over other alternatives as it presents higher sensitivity and biological resolution in bioinformatics analysis from sequenced *16S rRNA* amplicons compared to other analysis methods (Prodan et al., 2020). To achieve this goal, a custom designed pipeline analysis was employed (Supplementary Data). Briefly, amplification primers were eliminated from the reads, and a maximum of 120 nucleotides were trimmed from both reads’ ends if their quality was below Q30. After individual processing, overlapping forward and reverse reads were merged in paired-end sequencing datasets and chimeras were removed. Taxonomic assignment was then performed on the ASVs by means of the RDP Naive Bayesian Classifier algorithm using as reference the SILVA v. 138.1 database (McLaren and Callahan, 2021; Yilmaz et al., 2013). Those ASVs not classified at the taxonomic domain level, and/or singletons were excluded from subsequent analyses by using the tools included in the Phyloseq package in R (McMurdie and Holmes, 2013; v. 1.44.0) and following the curation process detailed in Johnson et al. (2021). By doing so, each study’s data was compiled in a single Phyloseq object that allowed managing all the ASVs obtained, despite the hypervariable regions of the *16S rRNA* sequenced. Resulting individual abundance tables were then merged into one single table, and different ASVs with total coinciding taxonomic assignments were unified in one single ASV.

### 2.3 Statistical analysis

All environmental data collected presenting different measurement units in the studies were unified to a single scale prior to statistical analyses (Supplementary Table S3). Nonparametric Wilcoxon test for two samples was employed to determine which environmental variables presented significant differences between subsurface and depth environments. Those that were found to be significantly different were included in subsequent analyses. Non-Metric Multidimensional Scaling (NMDS) with Bray-Curtis distances (Clarke, 1993) was then performed on those data to study the similarities of the samples based on their environmental settings.

Alpha diversity analyses by means of Shannon Index and Simpson Index were calculated with the vegan package (Dixon, 2003; v. 2.6.4) for R. Similarity Percentage Analysis (SIMPER) was then carried out using Bray-Curtis distances to look for the taxa that contributed the most to the dissimilarity between subsurface and depth samples. Subsequently, Indicator Species Analysis or ISA (Dufrêne and Legendre, 1997) was then performed to identify those taxa that were strongly associated with one group or another, employing R package indicspecies (De Cáceres et al., 2016; v. 1.7.14)

Canonical Correspondence Analysis (CCA) was also carried out to determine the presence of microbial population distribution patterns associated with gradients in the included environmental data.

## 3 Results

### 3.1 Physicochemical parameters

After applying the Wilcoxon test to the normalized values, significant differences were found for seven parameters: nitrate (p-value = 0.000061), nitrite (p-value = 0.000006), temperature (p-value = 0.037), pH (p-value = 0.0051), phosphate, magnesium (p-value = 0.023), calcium (p-value = 0.0043), depth (p-value < 2.2e^−16^) and salinity (p-value = 0.000065) (Supplementary Figure S2), when comparing subsurface and depth environments. The rest of the variables were discarded in subsequent analyses (Supplementary Figure S3).

**Figure 2:**
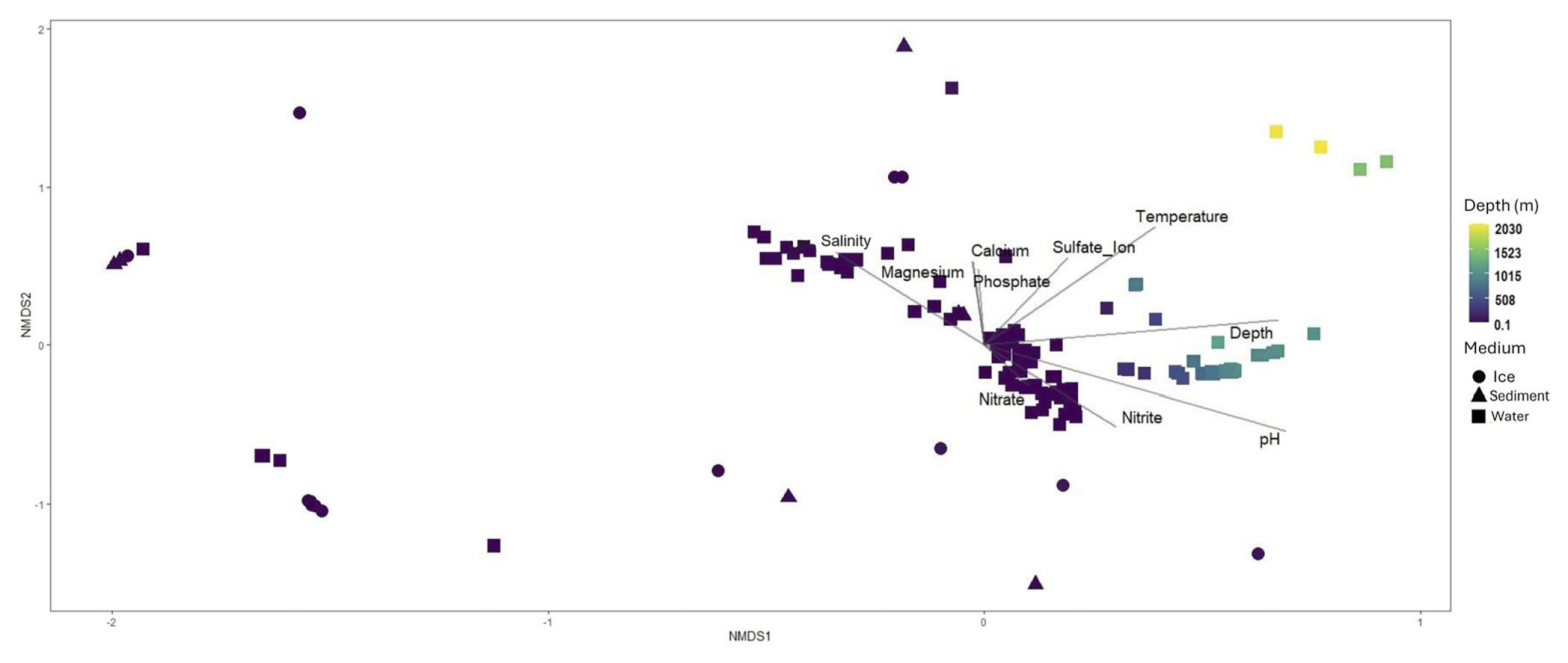
Non-metric Multidimensional Scaling or NMDS representation (stress = 0.1459). The shape of the points represented shows the type of matrix from where the samples were taken and the colors represent the depth. Vectors length represent the strength of correlation between the ordination and the variable, while its direction points into the steepest increase of its value.

NMDS analysis (Figure 2) showed a dispersion of a large portion of the subsurface samples located close to the center of the ordination, mainly influenced by the presence of nitrite and nitrate. The dispersion of most of subsurface samples appears to be due to the high salinity found at these samples, and, to a lesser extent, due to the concentration of calcium or phosphate. Conversely, the depth at which samples were taken split the distribution of those samples obtained at greater depths from the shallower ones, except for a group of the deepest samples which are located separately. pH seems to be the main driver for the grouping of the largest group of deep samples, while the deepest ones are mainly influenced by temperature and, to a lesser extent, sulfate ion.

### 3.2 Taxonomic composition and diversity indexes

**Figure 3:**
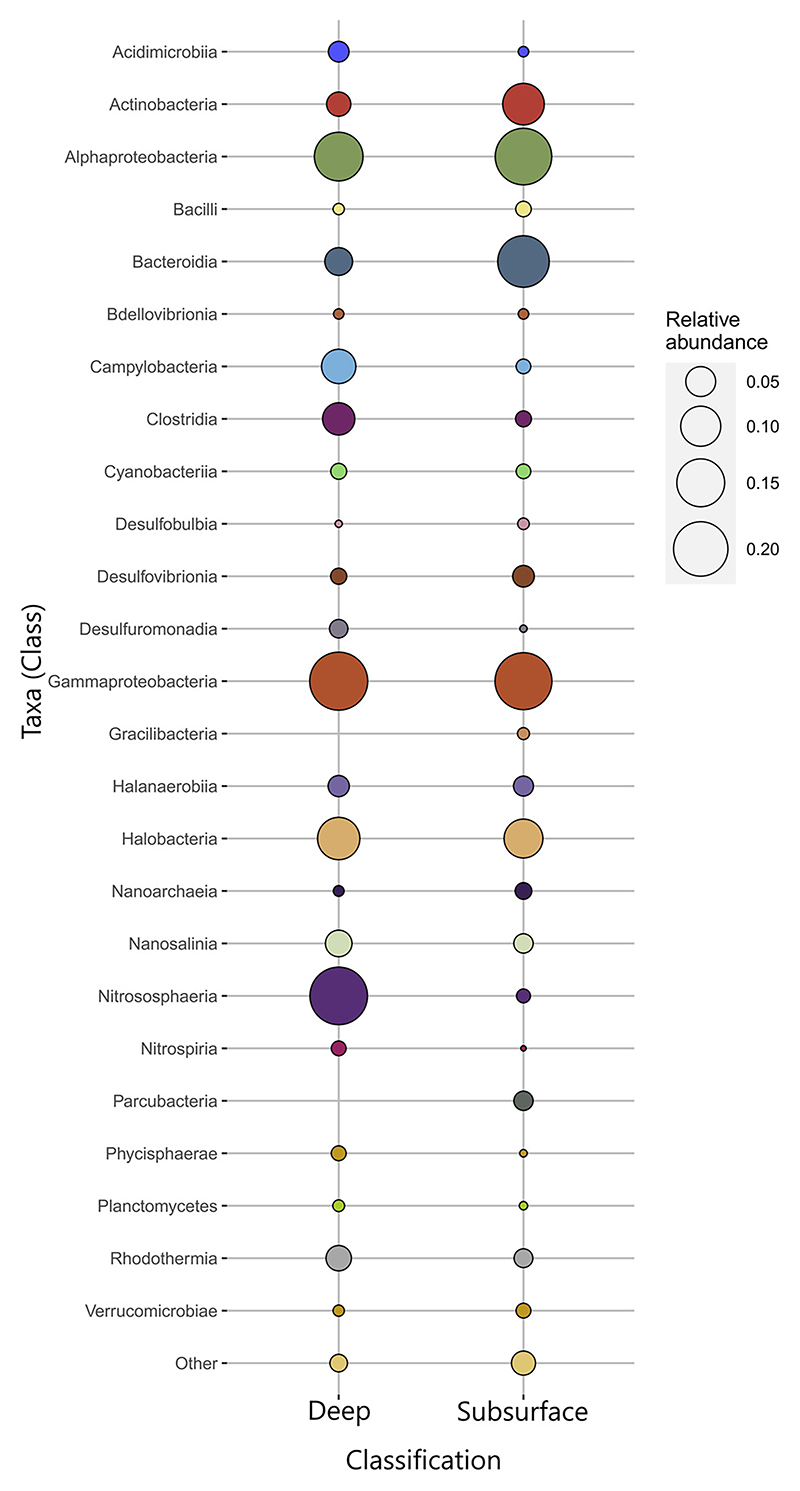
Bubble plot representing the relative abundances of the 25 most abundant class level taxa grouped by their depth (Subsurface or Deep). The “Others” taxa category includes all taxa (both Archaea and Bacteria) that were present in lower relative abundances.

The meta-analysis conducted denoted a higher relative enrichment of members of *Alphaproteobacteria* and *Gammaproteobacteria* classes in both subsurface and depth environments (Figure 3), followed by *Halobacteria* and *Halanaerobiia*. In the deep environments, taxa within classes such as Nitrososphaeria, Clostridia or Campylobacteria standed out for their relative abundance compared to that found in the subsurface samples, while the *Actinobacteria* and *Bacteroidia* classes are the ones with the highest representation in subsurface environments. Other taxa related to sulfur (such as *Desulfobulbia, Desulfovibronia* or *Desulfuromonadia*) were found in both environments. *Cyanobacteria* and *Bacilli* distribution also followed this pattern, despite deep and subsurface environments having different sunlight conditions.

**Figure 4:**
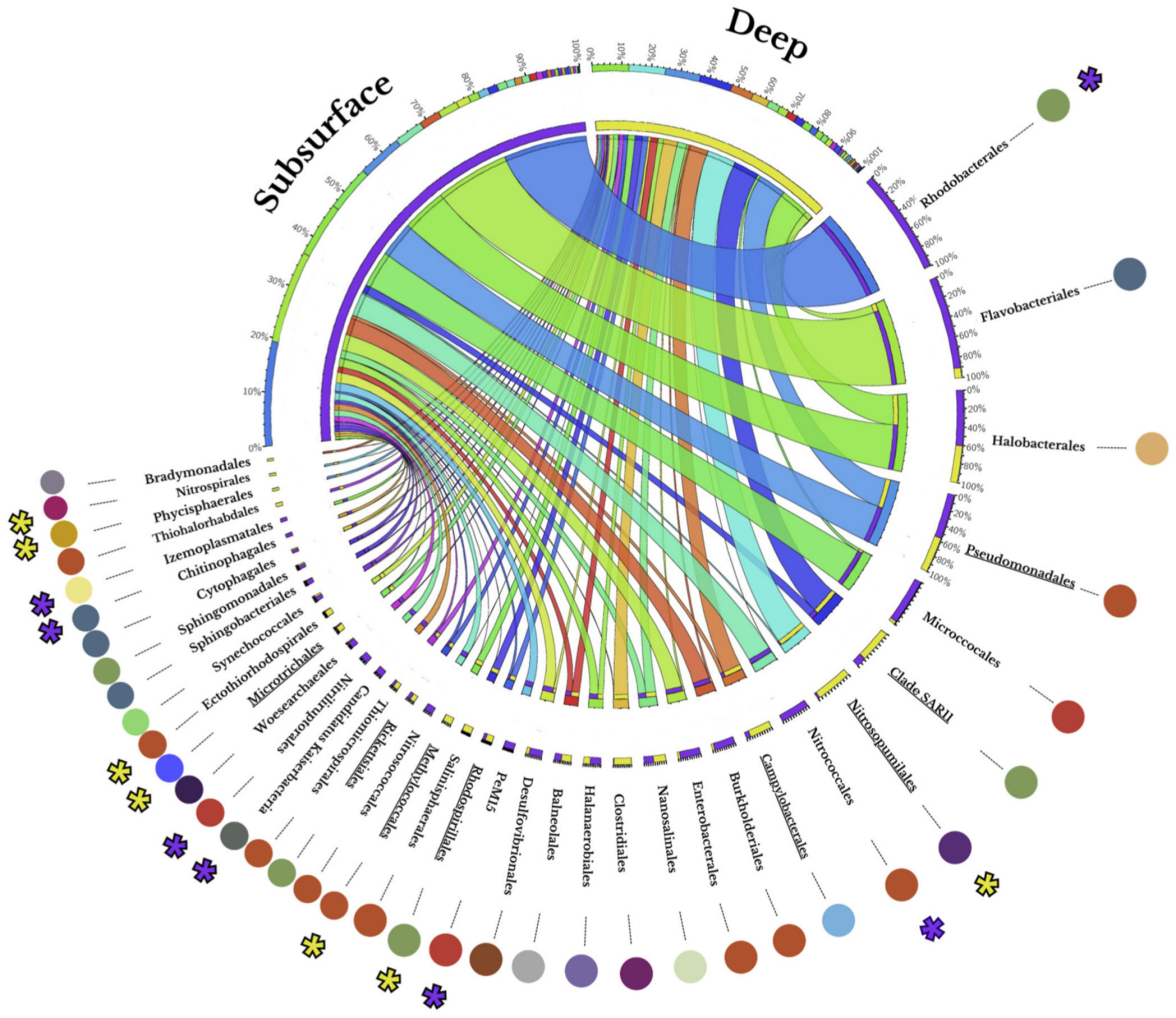
CIRCOS graph. Two columns are defined in different colors for each depth group (subsurface and deep). The ribbons (links crossing through the middle of the central circle) indicate the taxonomical orders within the 38 most abundant ones identified in the two sets of samples, while their width shows the relative abundance of each taxon in both types of environments. Colors under the Deep and Subsurface titles represent the percentage of samples composed of different taxa, following the same color pattern as the ribbons. The columns situated below the names of each taxonomical order represent the proportion of counts registered in deep (yellow) or subsurface environments (purple). Exterior circles linked by a thin line to an order name represent the class to which each order belongs, following the same color pattern as the bubble plot shown earlier in the manuscript. Finally, asterisks point out the orders that presented statistically significant (p-value < 0.05) differences for the IndVal analysis (their color representing the type of environment for where they were selected as potential “indicative species”). while underlined names were statistically significant for the SIMPER analysis.

To have more detailed information about which taxonomic groups contributed more to the differentiation of environments, IndVal (Indicator Value obtained from ISA) and SIMPER analyses were applied to order-level instead of only to classes data (Figure 4). These analyses resulted in 32 taxa with differential distributions between the two samples and/or a valid classification as indicator groups of one of the two environments. Seventeen of them were among the most represented orders, included in the classes *Actinobacteria, Alphaproteobacteria, Gammaproteobacteria, Nitrososphaeria, Phycisphaerae, Parcubacteria, Acidimicrobiia, Nitrospiria* and *Clostridia*. Further analysis of the taxonomic classification at order level showed that highest taxonomic representations corresponded to subsurface environments (Figure 4). Most archaeal orders (*Halobacterales, Nanosalinales, Halanaerobiales*) were found to be evenly distributed between both environments, except for members of the *Woesearchaeales* order (*Nanoarchaeia*), present exclusively in deep environments. Most predominant bacterial orders (*Rhodobacterales, Flavobacteriales*) belonged to subsurface environments, while the least present taxa (*Nitrospirales* and *Phycisphaerales*) were found exclusively in deep environments.

Shannon and Simpson biodiversity indices (Supplementary Figure S4) showed statistically significant higher mean values for Depth environments (Wilcoxon p-value < 0.05).

### 3.3 Multivariant statistical exploratory analysis

**Figure 5:**
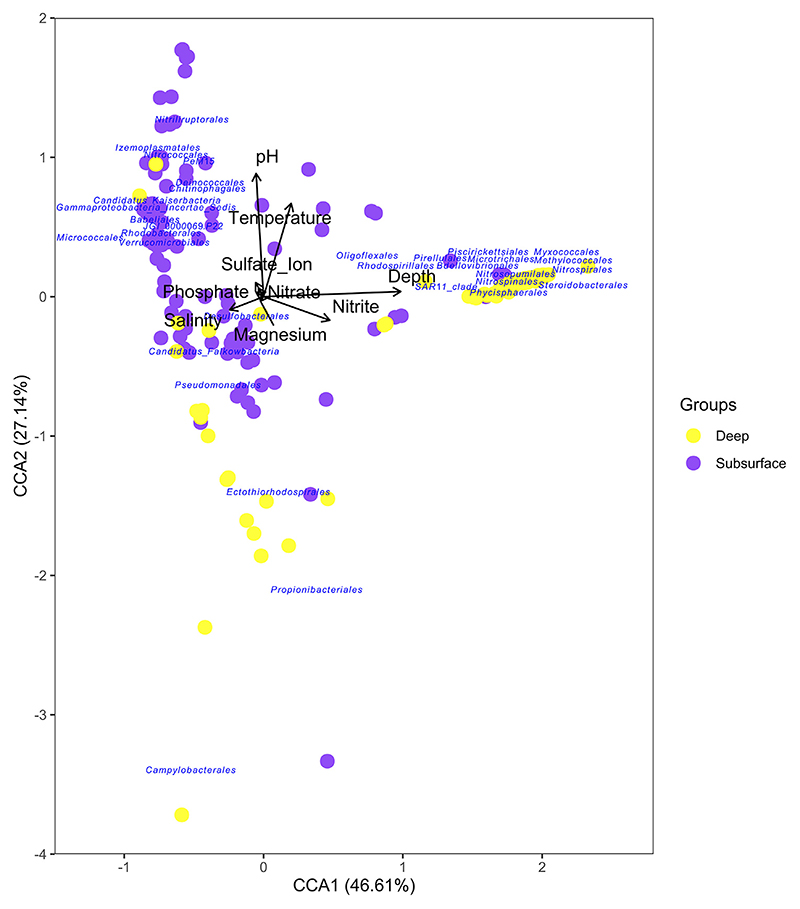
Canonical Correspondence Analysis (CCA) illustrating the relationships between environmental variables and the 33 significantly different taxa across all samples. Arrows represent the environmental variables, with their length indicating the increasing values for that variable and representing the strength of correlation with the species distribution. The CCA1 and CCA2 explain the 46.61% and the 27.14% of the total explained variance, respectively.

Canonical correspondence analysis (CCA) including the 33 orders with significant differential presence depicted by IndVal and SIMPER analyses identified the main environmental factors influencing microbial community composition at that taxonomical level. The total variance explained between the two axes was 73.75% (Figure 5). The distribution of subsurface samples is determined by temperature, pH, salinity, and the presence of phosphate and sulfate ions. Conversely, a large group of deep samples are separated from most of the others by magnesium and salinity, whose distribution is driven by the depth vector, representing the increasing distance from the surface.

In terms of the location of the taxa represented, two clusters of microbial orders were identified, which were divided along CC1. The first cluster formed around the vectors of sampling depth and nitrite concentration and included the following taxa: *Myxococcales, Methylococcales, Microtrichales, Phycisphaerales, SAR11 clade (Candidatus Pelagibacterales), Rhodospirillales, Bdelovibrionales, Piscirickettsiales, Nitrospirales, Nitrosopumilales, Nitrospinales*, and *Pirellulales*. The second cluster is related to pH, temperature, sulfate, nitrate and phosphate ions and groups the taxonomic orders of *Verrucomicrobiales, Izemoplasmatales, Nitrococcales, Chitinophagales*, or *PeM15* (uncultured *Actinobacteria* order), among others, coming mostly from subsurface samples. It is also noteworthy that two orders (*Ectothiorhodospirales*, and *Pseudomonadales*) appeared along CCA2 axis inversely related to pH, Temperature and the presence of sulfate or phosphate ions.

## 4 Discussion

### 4.1 Analog environments: understanding the environmental stressors and conditions

Given the wide diversity of analog environments on Earth that resemble those found on Icy Moons, it is essential to identify common adaptation patterns of microbial communities (i.e., specific metabolisms or protective cell structures) in order to better focus future search for signs of life. These adaptations are thus reflected in the generation of specific metabolites that would provide a reference as biomarkers to be found in exploratory missions. Therefore, the approach followed in this study enables the development of a more precise inventory of prospective biomarkers for future life-detection missions to Europa and Enceladus.

Firstly, our results point to subsurface environments being more influenced by hypersaline conditions than those existing at deeper points. This may be due to evaporation processes (Li et al., 2023) that are more frequent in regions directly related to external climate or to the presence of frozen matrices that determine the formation of brine channels with high values for salinity (Fox-Powell and Cousins, 2020; Petrich and Eicken, 2016). Additionally, under faint atmospheres in the Icy Moons, the irradiation of the ice layer by charged particles could provoke radiolytic reactions resulting in the breakdown of water molecules, thus leading to a decrease in pH (Weber et al., 2023; Teolis et al., 2017). These interactions would also lead to an increase in oxygen concentrations, which would then become bioavailable (Ray et al., 2020), thus creating diverse conditions for putative microorganisms to develop under polyextremophilic environmental conditions. As a result, the presence of oxygen might not be scarce in the upper layers of ice present in the Icy Moons, but it would decrease exponentially in deeper layers of ice and the ocean beneath. Following that, it is also worth mentioning that our results showed both nitrite and nitrate increase in lower salinity environments, such as the Depth ones. This has been linked to biological processes that may occur under low oxygen rates associated with hypersaline environments in previous studies (Shadrin, & Anufriieva, 2020).

### 4.2 Taxonomic and metabolic diversity

The presence of diverse metabolic sources in terrestrial analog environments to the Icy Moons sustains the diversification of ecological niche exploitation strategies by oligotroph microbial communities. Among the correlations observed between taxa and physicochemical variables, increasing pH levels were shown to have a possible effect on nitrate production rates (Kinh et al., 2016), reaching the highest accumulation values for this molecule in subsurface samples with the presence of *Nitrococcales* order and other alkaliphilic taxa such as *Nitriliruptorales* (Sorokin et al., 2009). Within *Nitrococcales* order, some genera (such as *Nitrococcus*, found in our samples, data not shown) can oxidize nitrite (Nitrite-Oxidizing Bacteria group) (Füssel et al., 2017). Thus, this kind of chemolithotrophic pathway is feasible under the described environmental conditions for subsurface environments and, by a syntrophic relation with, for instance, the identified *Nitrosococcales*, able to oxidize ammonia in subsurface environments. The alkaline pH along with salinity could explain the lack of other denitrifiying bacteria in subsurface environments that would complete the N cycle, as in alkaline hypersaline environments the nitrite reductase nirK, which is the main nitrite reductase under hypersaline conditions (Torregrosa-Crespo et al., 2023), reduces its abundance (Pan et al., 2023). Moreover, in these environments most of haloarchaeal denitrifiers (such as *Halobacterales*, identified in subsurface samples in this study) lacked a complete set of genes needed for the completion of this pathway (Miralles-Robledillo et al., 2021), proving that other groups potentially present would not be able to complete the nitrogen cycle.

Deeper ecosystems are shown to differentiate from subsurface ones by the accumulation of nitrite and nitrate. It is especially remarkable the high accumulation of nitrite, suggesting that the nitrogen cycle might not be completing, despite the presence of ammonia oxidation taxa such as *Nitrosopumilales, Phycisphaerales*, or *Nitrospinales*. Assuming there is no lack of data or inconsistent temporal sampling in the referenced studies, this accumulation could be interpreted as an adaptive strategy based on energy efficiency: as survival strategies in hypersaline environments (requiring the synthesis of compatible solutes or the extrusion of ions out of the cell against the gradient) are highly energy-demanding (Oren, 2016), metabolisms such as nitrification may not provide enough energy to sustain cell development.

In addition to nitrogen cycling, detected *Desulfobacterales* or *Pseudomonadales* orders, known to be directly related to the sulfur cycle in terrestrial environments (Borzenko, 2023), may explain the lack of sulfate ion in the samples. The reduction of this ion to other compounds like sulfide (H_2_S), one of the main metabolisms found in several terrestrial hypersaline environments (Borzenko, 2023), would be the primary reason for this absence. Therefore, this metabolism could be sustained in the deeper hypersaline zones of the oceans on Europa, where sulfur compounds and anoxic conditions are met. Moreover, the presence of methanotrophic organisms like *Methylococcales* in deep environments could be related to methane generation on the seafloor by geochemical processes such as serpentinization (Lecoeuvre et al., 2020). These reactions have been considered possible on Enceladus given the detection of dihydrogen in the southern hemisphere geysers and CO_2_ in the subsurface ocean (Affholder et al., 2022), serving as a potential metabolic basis under these conditions. Although these metabolisms could also occur on Europa given methane records (Weber et al., 2023), they might not be the most feasible strategies. This is because various sulfur compounds have been detected, ubiquitously present in both subsurface and deep environments (Bouquet et al., 2024; Weber et al., 2023; Trumbo et al., 2019), leading to the consideration of sulfur metabolism as the predominant metabolic basis.

Other types of metabolisms that are not that frequent under Earth’s conditions could be considered as potential metabolisms in extraterrestrial habitats. As an example, those related to the release of phosphate ion (a compound found principally in subsurface samples) could be occurring. *Desulfotomaculales* order, which oxidates phosphite in dissimilatory reactions (Ewens et al., 2021) has been identified in some analog samples considered in this study (data not shown), and the formation of phosphites under Enceladus conditions has been inferred (Postberg et al., 2023).

Some other organisms identified in this study, such as *Micrococcales, Clostridiales* or *Desulfobacterales*, may also present different feasible strategies for autotrophic anabolism carried out in Earth’s analog environments. Specific RUBISCO (Ribulose-1,5-bisphosphate carboxylase/oxygenase) adaptations enabling activity in cold, hypersaline permafrost brines are found in *Micrococcales* (Harrison et al., 2025), making the Calvin-Benson-Bassham cycle the most prevalent in our planet. These kinds of modifications suggest that this cycle could operate under Enceladus conditions where CO_2_ is robustly identified. A simpler alternative, the Ljungdahl pathway, uses metalloenzymes and cofactors and occurs abiotically in some hydrothermal vents (Belthle and Tüysüz, 2022). Given the potential serpentinization happening on Enceladus (Affholder et al., 2022), a partial Ljungdahl route in its deep ocean might be viable, as the resulting dihydrogen molecules could function as a reducing agent. The *Clostridiales* class, which correlated with deep environments in our results and can produce acetate (a pathway intermediate), could be a key focus for further studies. As for Europa, internal carbon sources (Trumbo and Brown, 2023) suggest this pathway could also operate within the moon’s ocean, possibly alongside sulfur oxidation or similar metabolisms that clades as *Desulfobacterales* are currently able to maintain here on Earth.

### 4.3 Biomarkers

In the following sections, the diversity of survival strategies present in the identified taxa will be discussed with the aim of narrowing down the biomolecules associated with their activity in order to guide future research efforts.

#### 4.3.1 Metabolites

**Figure 6:**
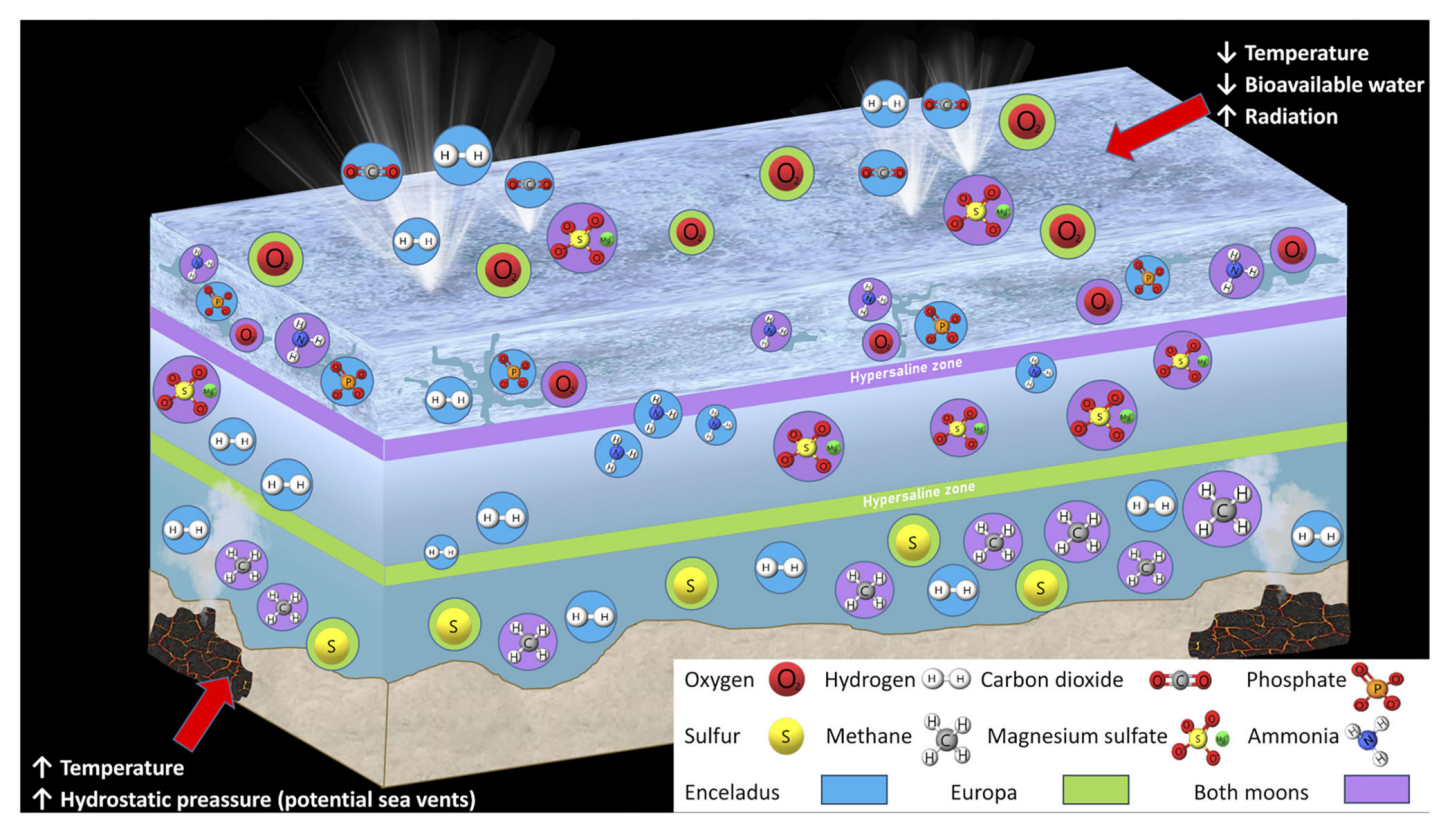
Environmental stressors and summary of the composition of the potential niches present in both Icy Moons.

Following recent findings on Europa, as well as on Enceladus, hypersaline environments may be widely present (Figure 6). This is the case for the ocean and the ice-water interface regions, as well as to channels within the ice on Europa (Khurana et al., 2009; Wolfenbarger et al., 2022), and the brine channels formed at the ice-water interface on Enceladus (Fox-Powell and Cousins, 2020). Thus, adaptation to these conditions must be crucial for the establishment of any putative organism. However, in polyextremophilic environments, multiple stressors challenge membrane stability, requiring diverse molecular adaptations to preserve cytoplasmic integrity. Thus, although “salt-in” strategies could develop alongside adaptations to other stressors, modifications related to multiple environmental pressures, such as lipid adjustments (Finkel et al., 2023) and the use of certain compatible solutes involved in the “salt-out” strategy would enable better survival under polyextremophilic conditions. While in deep regions the presence of molecules that contribute to withstand high hydrostatic pressures (piezolytes) is likely more common, groups associated with unstable subsurface environments must present adaptations to challenges like desiccation or climatic changes. The most widely distributed osmolyte synthesis among Bacteria is the amino acid derivative glycine beatine (McParland et al., 2021). This molecule preserves the cell’s structures against low temperatures (Li et al., 2021; Ma et al., 2017) and it is synthesized from glycine or choline through pathways present in the majority of *Alphaproteobacteria* and *Gammaproteobacteria* (McParland et al., 2021), both being present almost evenly in both subsurface and deep environments analyzed in this study. Additionally, several of the orders related to deep environments in our results, such as *Rhodospirillales, SAR11 clade*, or *Methylococcales*, utilize ectoine or 5-hydroxyectoine (Imhoff et al., 2020; Lanclos et al., 2023; Kadam et al., 2023); being one of the most abundant compatible solutes in halophiles (Salma et al., 2020), it is intended for protection not only against hypersalinity but also against high pressures, extreme temperatures, and anhydrobiosis states (Czech et al., 2018). In fact, psychrophilic and halophilic traits tend to be coexpressed in extreme cold environments, as salt can lower the freezing point of water enabling metabolic reactions to happen at subzero temperatures (Rampelotto, 2010; Garcia-Descalzo et al., 2020; Purwar and Srivastava, 2024). Furthermore, the combination between lipid modifications and these molecules is a strategy that currently happens in some piezophilic taxa that have been identified in this study such as *Colwelliaceae* (Enterobacterales) (data not shown, Peoples et al., 2020). These compounds, in areas closer to the surface where freezing conditions are prolonged/permanent, could enable the adoption of an anhydrobiotic state inside the cell (Czech et al., 2018), maintaining the viability of the processes contained within. Another osmolyte mainly present in Prokaryotes is the disaccharide sugar trehalose, being produced through different enzymatic routes (McParland et al., 2021), common in Actinobacteria, a recurrent taxon in this study (Figure 3). This disaccharide can act as a cryoprotector (Salma et al., 2020) by reducing the freezing point of the cytoplasm and stabilizing proteins, or even function as an energy storage in oligotrophic bacteria (Boysen et al., 2020), both probable environmental conditions in the sampling sites included in this study and in the extraterrestrial environments in the Icy Moons.

#### 4.3.2 Structural molecules

In addition to the metabolites mentioned above, there are lipid adaptations ubiquitously distributed in the membranes of some microorganisms, including halophilic archaea. Generally, the membrane composition of these organisms tends to have a higher proportion of double chains of isoprenoids linked to a glycerol molecule by ether bonds rather than ester bonds. This increases the stability of the plasma membrane, as the bonds are more resistant to hydrolysis (Finkel et al., 2023). Thus, the presence of lipid components such as archaeol can be associated with the taxa recorded in the study environments, such as *Nanosalinia, Halobacteria*, or *Halanoaerobiia*. These groups, ubiquitous in terrestrial hypersaline environments regardless of their depth, could therefore present adaptations that can be extrapolated to the conditions of the Icy Moons, and therefore studied in future missions.

Given that on Europa hypersalinity predictions could apply to both the ocean and the ice-water interface regions, as well as to channels within the ice (Khurana et al., 2009; Wolfenbarger et al., 2022), and to brine channels formed at the ice-water interface on Enceladus (Fox-Powell and Cousins, 2020), it is inferred that this type of adaptation must be key to establishing any biological structure under these conditions. Additionally, considering the multiple stress factors influencing the putative biological activity of the Icy Moons, lipid membrane stability would be a limiting factor not only for the functionality of biological systems but also for prebiotic systems. Therefore, lipids modified for hypersalinity, which are present in numerous taxa described in this study, are likely to be key components in the viability of lipid structures, making them strong candidates for use as biomarkers.

Additionally, the accumulation of compatible solutes within these structures would be a plausible strategy for withstanding the expected high hydrostatic pressures and hypersaline conditions in the different niches of these moons. The combination of lipid modifications and these molecules is a strategy that is currently observed in certain piezophilic taxa that have been identified in this study (data not shown) such as *Colwelliaceae* (Peoples et al., 2020). These compounds, in areas closer to the surface where freezing conditions are prolonged or permanent, could enable the adoption of an anhydrobiotic state inside the cell (Czech et al., 2018), thereby maintaining the viability of the processes contained within.

#### 4.3.3 Search and detection

The goal of this study is to expand the existing knowledge on possible biomarkers of microbial origin that could be detected in future space exploration missions aimed at finding signs of life. The comprehensive discussion above about different metabolites led us to differentiate between several types of potential biomarkers; therefore, the detection of these compounds using current technology should be carried out differentially. As mentioned before, the heterocycle ectoine, a widely present osmolyte (McParland et al., 2021) that could allow survival at different depths, could be excreted outside the cell in response to changes in osmotic pressure (Feng et al., 2023). This cellular strategy could therefore enable ectoine to be detected by transit spectroscopy, as it has been done before with other heterocyclic molecules (Puzzarini et al., 2017), in the outer layers of the ice and atmosphere (coming from the plumes). Among the rest of the above-mentioned compounds, there are also other examples of molecules that can also be transported extracellularly, such as glycine betaine, but their detection is nowadays more difficult due to their non-cyclic structure. Current methods (liquid chromatography coupled with mass spectrometry, LC-MS) would allow the detection of this type of osmolytes in large sample amounts, as has been demonstrated previously by detecting them directly from sea water (Airs and Archer, 2010). Portable systems for both techniques are currently being designed (Arevalo et al., 2019; Rahimi et al., 2020) and hopefully will allow the *in-situ* detection of a more diverse range of organic molecules in future missions. This would include other compounds related to metabolism, such as metalloproteins associated with autotrophic anabolism which can currently be detected in salt-rich matrixes using these techniques (Tang et al., 2022).

Finally, structural biomolecules that are stable under hypersaline conditions, such as archaeol, are also ubiquitously present in analog environments. These lipids also have highly recalcitrant hydrocarbon skeletons, which allow them to be detected even after billions of years (Finkel et al., 2023). This increases the likelihood of their detection under extraterrestrial conditions. In this context, is essential to prioritize the detection of complex biomolecules (lipids, proteins and nucleic acids; Fairén et al., 2020), as the interpretation of metabolic route intermediates or final products, such as acetate or methane, as biomarkers is still controversial (Thompson et al., 2022; Watanabe and Ozaki, 2024). Therefore, groundbreaking biobased technologies such as SOLID-LDChip instrument (Parro et al., 2011) or other Lab on a Chip (LoC) designs could provide a better performance and ease of transportation (Cinti et al., 2023) when developing specific sensors to detect biomolecules, including field studies (Sánchez-García et al., 2020; Fernández-Martínez et al., 2021).

## 5 Conclusion

The deep and subsurface environments potentially present in Europa and Enceladus impose multiple environmental stressors that could shape microbial communities, metabolic pathways and adaptative strategies. Terrestrial analogs demonstrate that microorganisms with polyextremophilic traits can persist across a range of subsurface and deep habitats, regardless of water availability or variations in radiation exposure. Moreover, enzymes involved in dissimilatory and assimilatory processes in both chemotrophic and organotrophic organisms can function under the elevated osmotic pressures found at different depths. The metanalysis presented here showed that for the case of Enceladus, methanotrophy enzymes coupled with those involved in the Ljungdahl route are more likely to be found in the deep ocean, while enzymes involved in nitrate and phosphite production could be present in the upper layers of water and ice, including the plumes. As for Europa, given the signs of strong tides beneath the ice, potential biomarkers associated with deep ocean environments could be found throughout the entire water column. This could apply to sulfur-related enzymes and Ljungdahl route metalloenzymes.

Although both moons possess tenuous atmospheres influenced by plume activity and ice-shell fractures, adaptations developed for survival in the deepest ocean layers may also confer resilience to low temperatures and desiccation conditions in the upper layers of ice. For both moons, a common survival pattern has been identified here: osmolytes and modified lipids are used as a shared adaptive strategy by taxa inhabiting their terrestrial analog environments, regardless of depth. These biomolecular adaptations provide cellular protection against the low temperatures and anhydrobiotic conditions present in plumes and ice layers, as well as against hypersaline conditions and high pressures that may occur in deeper ice strata and subsurface oceans.

Further characterization of the Icy Moons will refine the constraints on the plausible metabolic strategies, anabolic pathways, and survival mechanisms that can be inferred from the biodiversity described in this study. This will provide a robust framework for guiding future exploration for biosignatures in their deep and subsurface environments.

## Figures and Tables

**Table 1:** General information about the sets of samples used, their source, classification and main characteristics of interest for the meta-analysis.

Author/ Year	N° of samples used	Matrix	Classification	Range of depth (m)
Cooper et al. (2019)	17	Ice-water- sediment	Both	6.13–55
Fernández-Gómez et al. (2019)	11	Ice-water	Subsurface	0.85–1.6
Menéndez-Serra et al. (2021)	33	Water	Subsurface	0.1–0.4
Kalwasińska et al. (2018)	1	Water	Deep	405
Lach et al. (2023)	6	Water	Subsurface	125
Baricz et al. (2021)	34	Water	Subsurface	0.5–9
Andrei et al., 2015	8	Water	Subsurface	0.5–11
Akpolat et al. (2021)	4	Water	Both	1
Sisma-Ventura et al. (2022)	45	Water	Both	1–1045
Abdallah et al. (2014)	-	Water	Deep	1468–2030
Lee et al. (2014)	2	Water	Deep	845
Sierra et al. (2022)	9	Sediment-water	Both	0.35–0.75
Saxton et al. (2021)	3	Sediment-water	Deep	75.2–68

## Data Availability

The raw sequences analyzed in this study were retrieved from publicly available repositories and were originally generated in previously published studies; the corresponding source publications are comprehensively documented in the Supplementary Material of this article to facilitate traceability and access.

## Footnotes

**Supplementary material**
